# Quality of life among women with sexual dysfunction undergoing hemodialysis: a cross-sectional observational study

**DOI:** 10.1186/1477-7525-10-103

**Published:** 2012-08-31

**Authors:** Paulo Roberto Santos, José Roberto Frota Gomes Capote Júnior, Juliana Uchoa Cavalcanti, Cyntia Brito Vieira, Ana Rochelle Mesquita Rocha, Natália Alves Mineiro Apolônio, Elaine Barbosa de Oliveira

**Affiliations:** 1 Sobral School of Medicine, Federal University of Ceará, Avenida Comandante Meurocélio Rocha Ponte 100, CEP: 62.042-2800 Sobral, CE, Brazil

**Keywords:** Chronic kidney failure, Renal dialysis, Quality of life, Sexuality, Sexual dysfunction

## Abstract

**Background:**

Sexual function among women undergoing hemodialysis (HD) is under-studied and there is no consensus about the effect of sexual dysfunction (SD) on their quality of life (QoL). We aimed to determine the prevalence of SD and to compare QoL between women undergoing maintenance HD with and without SD.

**Methods:**

We included female end-stage renal disease (ESRD) patients undergoing HD during June 2011 in the only renal unit in the north of Ceará state, northeastern Brazil. The criteria for inclusion were age between 18 and 55, at least three months on dialysis and being sexually active. Women using antidepressant medication were excluded. We used the Female Sexual Function Index (FSFI), which evaluates six domains of sexual function, including desire, arousal, lubrication, orgasm, satisfaction and pain. The patients were classified as presenting SD if the total FSFI score was less than 26. For QoL evaluation, we used the validated Brazilian version of SF-36. This is a widely used 36-item questionnaire covering eight dimensions of QoL. Demographic data, time on dialysis, underlying etiology of ESRD, and laboratory measures were assessed in unit records.

**Results:**

Of a total of 58 women, 46 (79.3%) presented SD. There were lower scores related to physical functioning (48.2 vs. 71.2; p = 0.007), bodily pain (45 vs. 67.5; p = 0.010), vitality (52.1 vs. 69.1; p = 0.026) and social functioning (57.2 vs. 76.1; p = 0.034) among women with SD compared to women without SD. Physical functioning and role-physical presented positive linear correlation with FSFI scores, respectively, r = 0.322 (p = 0.013) and r = 0.345 (p = 0.007).

**Conclusion:**

The prevalence of SD among women on HD is very high, reaching nearly 80%. Women on HD with SD had worse QoL, especially physical aspects of QoL, when compared to women without SD. Therefore, approaches aiming to improve QoL among women undergoing HD should be considered.

## Background

End-stage renal disease (ESRD) is the stage reached by chronic renal diseases in which kidneys function is irreversibly lower than 15% of normal function. ESRD is fatal unless some kind of renal replacement is offered (dialysis or kidney transplantation). Worldwide there is a shortage of organs to transplant and new cases of ESRD are increasing rapidly, making hemodialysis (HD) the most used form of renal replacement [1]. There are more than one million patients in the world undergoing maintenance dialysis and nearly 90,000 in Brazil [2].

ESRD strongly affects sexual function, mainly because of endocrine abnormalities [3]. Sexual dysfunction (SD) is well studied among men mainly concerning erectile dysfunction. Erectile dysfunction is highly prevalent among ESRD men [4]. In our experience, erectile dysfunction is present in half of young men undergoing dialysis and it generally compromises mental health [5]. However, SD among women is less studied. For instance, a meta-analysis on SD found 21 studies comprising 4,389 men but only two studies covering 306 women [6].

Based on the few studies that have been conducted, it seems that SD is also common among women on HD [6]. However, based on a Brazilian study [7] and another from the United States [8], this apparently does not affect their quality of life (QoL), in contrast to many findings indicating that among male HD patients, the mental aspect of QoL is greatly affected by erectile dysfunction [5]. Do women under dialysis perceive SD in a way that their QoL is not affected? Is there a different pattern concerning QoL in men and women with SD on dialysis? These questions intrigued us, so we aimed to determine the prevalence of SD and to compare QoL between ESRD women undergoing maintenance HD with and without SD.

## Methods

### Sample

We included ESRD women undergoing HD during June 2011 in the only renal unit in the north of Ceará state, northeastern Brazil. The criteria for inclusion were age between 18 and 55, at least three months on dialysis and being sexually active. The 55-year age limit was based on a Brazilian study that found no sexual activity among female ESRD on dialysis older than 60 years [7]. Women using antidepressant medication were also excluded. Fifty-eight women were included from a total of 79. Among the patients excluded there were 10 with less than three months on therapy, 7 without sexual activity, 3 who refused to participate and 1 with cognitive impairment and another using antidepressant medication. All patients were undergoing conventional HD (three sessions of four hours per week) with polysulfone dialyzers (maximum number of reuses = 12). The study protocol and informed consent were approved by the ethics committee of Vale do Acaraú University, with which the hospital is associated.

### Evaluation of sexual dysfunction

We used the Female Sexual Function Index (FSFI) as validated in Brazil by Pacagnella et al. [9]. This is a 19-item questionnaire that evaluates six domains of sexual function including desire, arousal, lubrication, orgasm, satisfaction and pain. The overall score is the sum of the scores for each item multiplied by a domain factor. The minimum and maximum scores are respectively 2 and 36. Women with a score under 26 were classified as presenting sexual dysfunction. This cut-off was the same figure validated by other researchers [10].

### Measurement of quality of life

We used the validated Brazilian version of the Medical Outcomes Study 36-Item Short Form Health Questionnaire (SF-36) to measure the QoL level [11]. This is a well-validated 36-item questionnaire covering eight dimensions of QoL: physical functioning, role-physical, bodily pain, general health, vitality, social functioning, role-emotional and mental health. SF-36 generates scores from 0 (worst) to 100 (best) for each of the eight dimensions.

### Patient data

The demographic data, time on dialysis and underlying etiology of ESRD were assessed in unit records. The underlying kidney disease was classified by clinical criteria and not by histopathology. Classification of economic class was according to the criteria of the Brazilian Association of Research Institutes [12]. This validated instrument is used in marketing surveys and population censuses and grades economic class into five groups: A (highest economic class) through E (lowest). Its criteria include educational level of the head of household and ownership of household appliances.

The laboratory results were those routinely measured in HD patients: creatinine and albumin (markers of nutritional status and inflammation activity), hemoglobin (level of anemia, target among HD patients = 11-12 g/dl), calcium and phosphorus (calcium and phosphorus product above 55 mg^2^/dl^2^ indicates risk of tissue deposition) and Kt/V (index of the dose of dialysis delivered, target ≥ 1.2). Kt/V was estimated by using a second-generation Daugirdas formula [13].

### Statistical analyses

The Kolmogorov-Smirnov test was used to test for normal distribution of the continuous variables. Continuous variables with normal distribution are shown as mean ± SD, and if without normal distribution as median. Categorical variables are shown as percentages. Comparisons regarding continuous variables were performed by the Student-t and Mann-Whitney tests, respectively for continuous variables with normal and without normal distribution. Comparisons regarding categorical variables were performed by the Fisher test. Specifically, comparison between SF-36 scores according to SD was carried out by analysis of variance with covariates (ANCOVA), adjusted for age, economic class, time on dialysis, presence of diabetes, hemoglobin, albumin and Kt/V (which are traditional variables used in adjustments among HD samples). Correlation coefficients between SF-36 and FSFI scores were calculated by the Pearson test. P < 0.05 was considered significant. All the statistical analyses were performed using the Statistical Package for Social Sciences (SPSS, version 13.0 for Windows) [14].

## Results

Mean age of the sample was 38.9 ± 10.8 years. Participants were mainly from economic classes C (41.1%) and D (44.8%). Glomerulonephritis (56.9%) and hypertensive nephrosclerosis (20.7%) were the main causes of ESRD. Women were undergoing HD for a median of 36 months. Regarding laboratory measurements, mean values were: 8.3 ± 1.8 mg/dl for creatinine, 9.5 ± 2.0 g/dl for hemoglobin, 3.8 ± 0.3 g/dl for albumin, 46.8 ± 14.6 for calcium-phosphorus products, and 1.9 ± 0.3 for Kt/V index (Table 1).

**Table 1:** Comparison of sample characteristics between women with and without sexual dysfunction

**Variables**	**All sample**	**With sexual dysfunction**	**Without sexual dysfunction**	**P**
**Age**	38.9 ± 10.8	39.4 ± 11.3	35.9 ± 7.6	0.511
**Economic class**				
B	3 (5.2)	1 (2.4)	2 (16.6)	0.678
C	24 (41.4)	20 (43.4)	4 (33.3)	
D	26 (44.8)	21 (45.6)	5 (41.8)	
E	5 (8.6)	4 (8.6)	1 (8.3)	
**Time on dialysis** (months)	36	37.5	44	0.184
**Underlying kidney disease**				
Glomerulonephritis	33 (56.9)	25 (54.6)	8 (66.6)	0.834
Hypertensive nephrosclerosis	12 (20.7)	10 (21.7)	2 (16.8)	
Diabetic nephropathy	5 (8.6)	4 (8.6)	1 (8.3)	
Lupus nephritis	2 (3.5)	2 (4.3)	0	
Obstructive uropathy	2 (3.5)	2 (4.3)	0	
Polycystic kidney	1 (1.7)	0	1 (8.3)	
Undetermined	3 (5.1)	3 (6.5)	0	
**Laboratory**				
Creatinine (mg/dl)	8.3 ± 1.8	8.3 ± 1.8	8.5 ± 1.8	0.708
Hemoglobin (g/dl)	9.5 ± 2.0	9.4 ± 2.0	9.7 ± 1.7	0.650
Albumin (g/dl)	3.8 ± 0.3	3.8 ± 0.3	3.8 ± 0.4	0.951
Calcium-phosphorus product	46.8 ± 14.6	46.2 ± 14.9	49.3 ± 13.6	0.513
(mg^2^/dl^2^)				
Kt/V	1.9 ± 0.3	1.9 ± 0.3	1.9 ± 0.4	0.966

Data are means ± standard deviations and percentages (%) in parentheses.

Time on dialysis was not normally distributed and is shown as median.

From a total of 58 women, 46 (79.3%) presented sexual dysfunction. The median of FSFI scores was 15.4 (min = 2; max = 36) for the total sample, 6.8 for women with SD and 28.5 for those without SD. The scores according to each domain of sexual function are shown in Table 2. There were no differences regarding sample characteristics between women with and without SD, as shown in Table 1. There were lower scores related to physical functioning (48.2 vs. 71.2; p = 0.007), bodily pain (45 vs. 67.5; p = 0.010), vitality (52.1 vs. 69.1; p = 0.026) and social functioning (57.2 vs. 76.1; p = 0.034) among women with SD when compared to women without dysfunction (Table 3). Physical functioning and role-physical presented positive linear correlation with FSFI scores, respectively of r = 0.322 (p = 0.013) and r = 0.345 (p = 0.007) (Table 4).

**Table 2:** Comparison of sexual function domain scores between women with and without sexual dysfunction

**Domain**	**All sample**	**With sexual dysfunction**	**Without sexual dysfunction**	**P**
**Desire**^**a**^	2.5 ± 1.1	2.2 ± 1.0	3.8 ± 0.7	<0.001
**Arousal**^**b**^	1.9 ± 1.8	1.2 ± 1.1	4.4 ± 0.3	<0.001
**Lubrification**^**b**^	2.2 ± 2.1	1.5 ± 1.8	4.9 ± 1.0	<0.001
**Orgasm**^**b**^	2.2 ± 2.1	1.5 ± 1.8	4.9 ± 0.7	<0.001
**Satisfaction**^**c**^	3.1 ± 2.1	2.4 ± 1.8	5.6 ± 0.4	<0.001
**Pain**^**b**^	2.3 ± 2.2	1.6 ± 1.8	5.0 ± 0.9	<0.001

Data are means ± standard deviations.

^a^Range of scores 1.2-6.

^b^Range of scores 0-6.

^c^Range of scores 0.8-6.

**Table 3:** Comparison of SF-36 scores according to sexual dysfunction

**SF-36 dimensions**	**With sexual dysfunction**	**Without sexual dysfunction**	**P**
**Physical functioning**	48.2 ± 26.3	71.2 ± 21.7	0.007
**Role-physical**	20.6 ± 31.3	37.5 ± 34.5	0.054
**Bodily pain**	45.0 ± 27.1	67.5 ± 22.9	0.010
**General health**	42.0 ± 23.8	46.0 ± 21.9	0.606
**Vitality**	52.1 ± 23.3	69.1 ± 22.2	0.027
**Social functioning**	57.2 ± 27.6	76.1 ± 23.4	0.034
**Role-emotional**	28.0 ± 37.0	52.8 ± 48.1	0.057
**Mental health**	56.3 ± 23.3	70.6 ± 19.5	0.056

Data are means ± standard deviations.

**Table 4:** Correlation coefficients between SF-36 and FSFI scores

**SF-36 dimensions**	**FSFI scores**	
	**R**	**P**
**Physical functioning**	0.322	0.013
**Role-physical**	0.345	0.007
**Bodily pain**	0.112	0.402
**General health**	0.113	0.398
**Vitality**	0.172	0.195
**Social functioning**	0.212	0.110
**Role-emotional**	0.215	0.105
**Mental health**	0.188	0.157

## Discussion

The SD prevalence of 79.3% is near the 80% found by Seethala et al. [8] and is within the range of 30 to 80% found in the overall literature [6]. The hypothesis that women with SD do not have low QoL was rejected, unlike found by other authors [7,8]. The results from our sample indicate that physical dimensions of QoL are lowered, mainly the physical aspects, based on the fact that physical functioning and bodily pain were the most affected dimensions among the women studied. Additionally, reinforcing the association between physical dimensions of QoL and SD, the only two dimensions linearly and positively correlated to FSFI scores were physical functioning and role-physical. The positive correlation between role-physical and FSFI scores is corroborated by a recent study [15]. This same study also found no correlation between mental health and SD among women on dialysis, in line with our results [15].

This is a completely different result from that we found in a sample of men with erectile dysfunction from the same renal unit in a previous study [5]. In that study, men undergoing hemodialysis with erectile dysfunction presented lower mental health, with no impairment of physical dimensions of QoL [5]. It is beyond the scope of the present study to clarify the mechanisms involved in the association between SD and physical dimensions of QoL among women. But we can suggest that physical aspects of QoL are largely dependent on the clinical physical status of patients, as in several other illnesses [16]. Unfortunately, the two studies [7,8] that found no correlation of SD with QoL among women on HD used a different tool to measure QoL, making it impossible to compare the present result on female patients’ physical QoL with the findings of those studies.

Since this is a cross-sectional study, we cannot reach any conclusions about the direction of the relationship: whether low physical QoL causes SD or SD causes low Qol. In any event, based on these preliminary findings it is advisable to consider SD as a target when planning interventions to improve QoL among women on dialysis. Unfortunately, there are few studies about treatment for SD among women on dialysis. The Cochrane database of systematic reviews contains 16 studies on interventions for treating SD in patients with chronic kidney disease, but only one of these covers women [17]. Possible approaches to female SD are hormone replacement (estrogen/progesterone and androgens), correction of anemia, adequate dialysis delivery and screening for depression [3]. In our sample, dialysis delivery reached the target (mean Kt/V of 1.9, target > 1.2), but anemia control must be improved (mean hemoglobin of 9.5 g/dl, target of 10-11 g/dl). Additionally, kidney transplantation must be encouraged because there are findings of improvement of SD after transplantation [18-20]. However, there are also divergent results showing SD of 93.4% among women receiving transplants [21].

We must emphasize that our sample is characteristic of a low-income area: more than half of the women belong to the lower economic classes, with few diabetics. Besides the impossibility of extrapolating our results to women in more developed regions, there are several other limitations. First, it is small sample from a single renal unit. Second, we did not evaluate medications used that could interfere with SD (except data on antidepressant medication), although a study reported no correlation between degree of sexual dysfunction and medication [22]. Third, we did not distinguish the marital status of the women, while Seethala [8] did address this issue, finding no correlation between SD and the marital status.

## Conclusions

For clinical practice, our finding of high prevalence of SD and its association with lower QoL points to the need for routine screening of sexual function among women undergoing HD, aiming to put into practice possible approaches to SD in an attempt to improve patients’ QoL, which is the main outcome of HD treatment. Future research should investigate larger samples, first to confirm our rejection of the hypothesis that QoL level is the same between women with and without SD, as found by others, and, second to determine risk factors for SD.

## Abbreviations

ESRD: end-stage renal disease; FSFI: Female Sexual Function Index; HD: hemodialysis; QoL: quality of life; SD: sexual dysfunction; SF-36: the Medical Outcomes Study 36-Item Short Form Health Questionnaire.

## Competing interests

The authors declare they have no competing interests.

## Authors’ contributions

PRS conceived and designed the study, analyzed the data and wrote the manuscript. JRFGCJ participated in the conception of the study and coordinated the collection and arrangement of the data. JUC, CBC, ARMR, NAMA and EBO were involved in collection and arrangement of the data.

## Author’s information

PRS, MD/PhD, works in Brazil at the Federal University of Ceará as associate professor and coordinates the renal unit of Santa Casa de Sobral Hospital.
